# Factors Associated with Vaccine Breakthrough Incidence among Health Care Workers Vaccinated with Inactivated SARS-CoV-2 Vaccine (CoronaVac)

**DOI:** 10.34172/jrhs.2022.86

**Published:** 2022-07-11

**Authors:** Muhammad Anshory, Cesarius Singgih Wahono, Mirza Zaka Pratama, Perdana Aditya Rahman, Aditya Satriya Nugraha, Ayu Sekarani

**Affiliations:** ^1^Allergy and Immunology Division, Department of Internal Medicine, Faculty of Medicine Universitas Brawijaya-Saiful Anwar General Hospital, Malang, Indonesia; ^2^Rheumatology Division, Department of Internal Medicine, Faculty of Medicine Universitas Brawijaya-Saiful Anwar General Hospital, Malang, Indonesia

**Keywords:** COVID-19, Healthcare workers, Hospital, Vaccination, Vaccine breakthrough

## Abstract

**Background:** Healthcare workers (HCWs) run a high risk of severe acute respiratory syndrome coronavirus 2 (SARS-CoV-2). The HCWs are prone to the SARS-CoV-2 infection in the hospital despite being fully vaccinated. The present study aimed to address the factors associated with the coronavirus disease 2019 (COVID-19) vaccine breakthrough among HCWs.

**Study Design:** A prospective cohort study.

**Methods:** Participants were 184 HCWs receiving two doses of inactivated SARS-CoV-2 vaccine (CoronaVac, Sinovac Life Science). All participants were followed for six months. Confirmed COVID-19 was defined as positive SARS-CoV-2 by reverse transcription-polymerase chain reaction (RT-PCR). Before undergoing RT-PCR, questionnaires were used to obtain information on demographic characteristics, profession, contact with COVID-19 cases, personal protective equipment (PPE), health protocols adherence, exercise, and nutritional habits.

**Results:** A number of 57 (31%) participants were COVID-19 positive. Close contact with COVID-19 cases (adjusted RR 6.82, 95% CI: 1.97, 47.98, *P*=0.044), being a resident doctor (adjusted RR 4.72, 95% CI: 1.11, 20.11, *P*=0.036), improper mask-wearing (adjusted RR 2.36, 95% CI: 1.15, 4.85, *P*=0.019), and lower frequency of eating fruit and vegetables (adjusted RR 2.73, 95% CI: 1.34, 5.57, *P*=0.006) increased the risk of vaccine breakthrough. Compared to single surgical masks, KN95 and N95 significantly reduced the risk of COVID-19 (adjusted RR 0.27, 95% CI: 0.07, 0.97, *P*=0.045 and adjusted RR 0.25, 95% CI: 0.07, 0.87, *P*=0.029), respectively.

**Conclusion:** As evidenced by the obtained results, being a resident doctor, close contact with confirmed COVID-19 cases, health protocol incompliance, as well as the lower frequency of fruit and vegetable consumption were associated with the risk of vaccine breakthrough among HCWs. Appropriate strategies are needed to prevent the risk of SARS-CoV-2 infection among HCWs.

## Background

 Coronavirus disease 2019 (COVID-19) is already announced by the World Health Organization (WHO) as a pandemic caused by the severe acute respiratory syndrome coronavirus 2 (SARS-CoV-2). COVID-19 cases started as an increasing trend in various nations. Until 12 June 2022, WHO has confirmed 534 213 703 cases of COVID-19, including 6 306 423 deaths. Indonesian government reported 6 058 736 confirmed COVID-19 cases, 156 635 deaths, and 4 091 101 recovered cases from 510 districts across 34 provinces.^1^ In order to stop the spread of SARS-CoV-2 infection, vaccination programs are expanding rapidly in several countries. In January 2021, the Indonesian government issued an emergency use authorization (EUA) for the inactivated SARS-CoV-2 vaccine, CoronaVac (Synovac Life Science, Ltd.). The priority group for CoronaVac vaccination is healthcare workers (HCWs) since they are at higher risk of SARS-CoV-2 infection at work.^2^ Until November 2021, 76 191 677 Indonesian people had been vaccinated completely, while the other 462 727 442 received their first dose.^3^

 The risk of infection may still exist in fully vaccinated people. Currently, most SARS-CoV-2 infections occur in individuals who have not been completely vaccinated; nonetheless, vaccination breakthrough cases have been reported in several hospitals worldwide.^4-6^ The Centers for Disease Control and Prevention (CDC) defined “vaccine breakthrough infection” as an infection of a fully vaccinated person. While another previously conducted study referred to breakthrough infection as SARS-CoV-2 infection after 14 days or more after the second dose of COVID-19 vaccine.^5^ Vaccine breakthrough is occurring in a small percentage of vaccinated individuals in the United States, observed in both clinical trials and observational settings. Breakthrough infections can help us understand the efficacy of vaccines against SARS-CoV-2. Evidence on breakthrough risk can inform public health policies, including recommendations of additional primary doses.^5^

 The previous national-based cohort in Chile demonstrated that the CoronaVac effectiveness was 65.9% in the prevention of COVID-19 infection.^7^ As of April 30, 2021, CDC recorded 10 262 SARS-CoV-2 vaccination breakthrough infections in 46 states and territories.^8^ The higher risk of vaccine breakthroughs in the HCWs is due to continuous exposure to SARS-CoV-2 in the hospital.^9^ Despite the high vaccine breakthrough incidence, few studies observed the risks associated with vaccine breakthroughs in HCWs. In light of the aforementioned issues, the present study aimed to assess the several risk factors associated with the incidence of vaccine breakthrough in HCWs who were already fully vaccinated with CoronaVac. By observing these risk factors, we hoped that this data could provide several preventive measures against the spread of COVID-19 infection in HCWs when working at the hospital.

## Methods

###  Study design and participants

 This prospective cohort study was conducted in Saiful Anwar General Hospital, Malang, Indonesia, from January to September 2021. The participants of this study were HCWs, enrolled by the following criteria: (1) the age range of 18-59 years old, (2) doctor or nurse who worked in the hospital, (3) receiving two intramuscular injections of CoronaVac vaccine (Sinovac Life Science, Beijing, China) within the interval of 14 days, each injection containing 3 µg/doses or equal to 600 SU inactivated SARS-CoV-2 virus. Doctors were assigned to two groups: (1) the specialists who worked as consulting physicians and did not directly attend to COVID-19 patients, and (2) the resident doctors who directly treated COVID-19 under the supervision of specialists.

 The exclusion criteria entailed: (1) pregnancy and breastfeeding, (2) an unstable condition due to some comorbidities (e.g., flare or uncontrolled autoimmune disease, history of anaphylactic reaction due to vaccination, asthma attack, unstable heart failure, or acute complications of diabetes), (3) a severe liver or renal impairment, (4) previous recovery from COVID-19 in less than three months before undergoing vaccination. The ethical committee approved this study (Ethical approval number: 400/050/K.3/302/2021). All the participants signed written informed consent before participation in the study.

###  Follow-up and confirmation of COVID-19 

 The participants were followed and observed through an electronic-based questionnaire since they had their two doses of CoronaVac injections on a monthly basis for six months to find the clinical symptoms associated with SARS-CoV-2 infection. If any of the participants had clinical suspicion of COVID-19 before the monitoring date, they could contact the examiner to fill out the questionnaire at that time. The clinical symptoms of suspected COVID-19 were as follows: fever (body temperature recorded above 38°C or subjective fever), nausea, cough (dry or productive), shortness of breath, chest pain or tension, fatigue or malaise, sore throat, headache, nasal discharge, constipation, muscle pain, nausea or vomiting, diarrhea, stomach pain, smell or taste changes, loss of appetite, as well as red or bruised toes or legs. The subjects with these symptoms underwent pharyngeal swabs for the real-time reverse transcription-polymerase chain reaction (RT-PCR) examination after the completion of the questionnaire on the same day. The confirmed case of COVID-19 was defined as the positive results of SARS-CoV-2 from the respiratory specimen by RT-PCR. The confirmed cases of COVID-19 were classified as having asymptomatic, mild, moderate, severe, or critical illness according to the National Institutes of Health (NIH).^10^ All participants who did not have any COVID-19 symptoms also underwent the swabs and RT-PCR examinations at the end of the month every month, despite being asymptomatic. The presence of vaccine breakthrough was defined if there was an incidence of COVID-19 infection confirmed by positive RT-PCR examination at least 14 days after the administration of vaccine.^5^

###  Data collection

 On the same day before undergoing pharyngeal swab examinations, participants were administered an electronic-based demographic questionnaire to obtain data about demographic characteristics (age, gender, presence of any comorbidities, profession, and the working unit), a history of contact with the confirmed COVID-19 patients within 14 days before undergoing the RT-PCR examination (presence of close contact, place of contact with the patient [at hospital, home, or neither], and a history of treating confirmed COVID-19 patients). The number and types of personal protective equipment (PPE) used when not treating COVID-19 patients in the hospital, type of masks worn when not treating the COVID-19 patients, adherence to the health protocols, exercise, and nutritional habits were also recorded.

 Adherence to health protocols was assessed by asking participants about the observance of social distancing (at least two meters), washing hands using soap or sanitizers, and wearing a mask when not treating COVID-19 patients in the last 14 days. The answers were categorized into the following criteria: (1) often (always complied with health protocols every day without being absent), (2) sometimes (if participants were absent from complying with the health protocols at least one day in a week), and (3) never (if participants did not comply to the health protocols at all). Moreover, the participants were asked about doing regular physical activity and its duration. In addition, their nutritional habit was assessed by asking them about meal frequency throughout the day and the consumption of fruits and vegetables in the last 14 days.

###  Statistical analysis

 Numerical data were displayed as mean ± SD if normally distributed; otherwise, they were presented as median (interquartile range). Categorical data were illustrated as frequency rates and percentages. The association between the risk factors and the COVID-19 infection after vaccination was observed using the univariate and multivariate logistic regression analysis. It was demonstrated as crude and adjusted (adjusted for age, gender, and presence of comorbid) relative risks (RR) with a 95% confidence interval (CI). Variables were not included in the multivariate model if their *P* value in univariate analysis was > 0.05 since they would contribute very little to the model performance. All statistical analyses were performed in SPSS software (version 25.0), and a *P* value less than 0.05 was considered statistically significant.

## Results

###  Characteristics of the subjects

**Figure 1:**
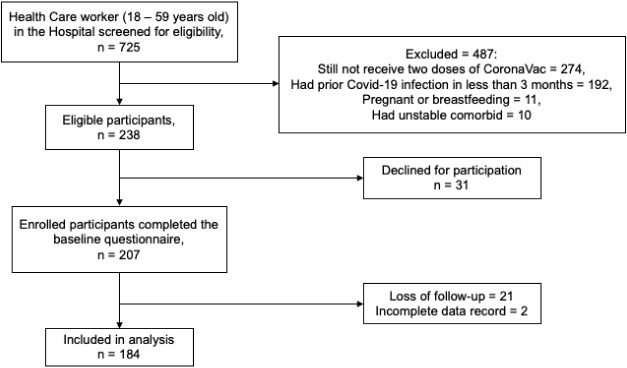
flowchart of the participants in the study.

 The flowchart of the participants enrolled in this study is depicted in Figure 1. A total of 725 HCWs in Saiful Anwar General Hospital, Malang, Indonesia, aged 18-59 years old, were screened for eligibility. Among this population, 238 participants met the inclusion criteria for the study (487 participants were excluded since they did not receive two doses of CoronaVac, had prior COVID-19 infection in less than three months, were pregnant, breastfed, or had unstable conditions). A number of 31 subjects withdrew from the study and did not complete the baseline questionnaire. Therefore, 207 health care workers were enrolled in the study and followed up for six months. Nonetheless, only 184 participants were included in the analysis since 21 cases were lost of follow-up and 2 subjects had an incomplete data record.

 The characteristics of the participants are displayed in Table 1. The participants’ mean age was 35.4 ± 9.0 years (ranging from 26-58 years). According to the participant’s profession, most subjects (67.4%) were resident doctors, while 14.7% and 17.9% of cases were specialist doctors and nurses, respectively. During the six-month follow-up, 57 (31%) participants were confirmed for COVID-19 by RT-PCR examination. The majority of subjects were positive for COVID-19 in the fifth month after the second CoronaVac injection. Among the 57 participants who had the SARS-CoV-2 infection, most were asymptomatic (29.8%) and had a mild degree of COVID-19 disease (64.9%). Only three subjects had a moderate infection (5.3%), while none had a severe disease.

**Table 1:** Characteristics of the Participants (n = 184)

**Variables**	**Number**	**Percent**
Gender		
Male	80	43.5
Female	104	56.5
Profession		
Specialist doctor	27	14.7
Nurse	33	17.9
Resident doctor	124	67.4
Comorbidities		
Not present	99	55.0
Present	85	45.0
Confirmed COVID-19		
Positive COVID-19	57	31.0
Negative COVID-19	127	69.0
Time of being confirmed COVID-19		
First month after vaccination	5	8.9
Second month after vaccination	1	1.8
Third month after vaccination	0	0.0
Fourth month after vaccination	5	8.9
Fifth month after vaccination	41	69.9
Sixth month after vaccination	5	8.9
Degree of COVID-19 infection		
Asymptomatic	17	29.8
Mild degree	37	64.9
Moderate degree	3	5.3
Severe degree	0	0.0

Table 2 illustrates the univariate analysis of the association of patients’ demographic data, including age, gender, comorbidities, profession, and the risk of contracting COVID-19 infection after vaccination. Age, gender, and presence of comorbidities were not significantly associated with the risk of COVID-19 infection according to the univariate logistic regression analysis. Nonetheless, compared to specialists as the reference, the resident doctors had an increased risk of contracting COVID-19 disease after vaccination (crude RR 3.42, 95% CI: 1.24-9.45, *P* = 0.018 and adjusted RR 4.72, 95% CI: 1.11, 20.11, *P* = 0.036).

**Table 2:** Univariate analysis of the association of the risk of COVID-19 infection after vaccination with demographics, profession, history of contact, and type of personal protective equipment (PPE) used in hospital

**Variables**	**Frequency**	**Crude**		**Adjusted**	
		**RR (95% CI)**	***P*** ** value**	**RR (95% CI)**	***P *** **value**
Age (mean ± SD)	35.4 ± 9.0	0.97 (0.93, 1.01)	0.103	0.97 (0.93, 1.02)	0.201
Gender					
Male	80	1.00			
Female	104	0.98 (0.52, 1.83)	0.944	0.99 (0.51, 1.96)	0.986
Comorbid					
Without comorbid	99	1.00			
With comorbid	85	1.56 (0.82, 2.98)	0.175	1.39 (0.70, 2.75)	0.349
Profession					
Specialist doctor	27	1.00			
Nurse	33	2.68 (0.95, 7.53)	0.062	3.15 (0.70, 14.14)	0.134
Resident doctor	124	3.42 (1.24, 9.45)	0.018	4.72 (1.11, 20.11)	0.036
Contact with COVID-19 patients					
Not contact	23	1.00			
Contact	161	3.85 (1.01, 13.45)	0.035	6.05 (1.16, 31.42)	0.032
Place of contact					
Hospital	125	1.00			
House	25	10.28 (1.74, 60.90)	0.010	21.37 (1.91, 239.72)	0.013
Outside of house / hospital	11	8.82 (1.79, 43.47)	0.007	14.16 (1.53, 131.13)	0.020
Treating COVID-19 patients					
No	103	1.00			
Yes	81	1.10 (0.57, 2.06)	0.771	0.94 (0.44, 2.00)	0.868
Number of PPE used in hospital	4 (3-6)	0.94 (0.79, 1.15)	0.473	0.94 (0.78, 1.12)	0.472
Type of Mask					
Surgical mask	13	1.00			
Surgical + cloth mask	33	0.56 (0.15, 2.03)	0.376	0.49 (0.13, 1.84)	0.291
KF94 mask	6	0.17 (0.02, 1.91)	0.151	0.14 (0.01, 1.60)	0.114
KN95 mask	50	0.28 (0.08, 0.99)	0.048	0.27 (0.07, 0.97)	0.045
N95 mask	82	0.29 (0.09, 0.97)	0.044	0.25 (0.07, 0.87)	0.029

 The univariate analysis for the association of exposure to covid-19 cases and the PPE usage in hospitals with the risk of COVID-19 infection is also shown in Table 2. Among 184 HCWs, 161 subjects (87.5%) were documented to contact confirmed COVID-19 patients at least 14 days before undergoing the RT-PCR examination. Contact with confirmed COVID-19 patients turned out to be associated with an increased risk of COVID-19 infection. Most subjects (78.1%) were exposed in the hospital, while 15.6% and 6.2% of cases came into contact at home and outside, respectively. Compared to those who had contact with covid-19 cases in the hospital, exposure to confirmed COVID-19 cases outside the hospital or house was linked to an increased risk of contracting COVID-19 infection.

 Not all HCWs who participated in this study treated COVID-19 patients, and only 81 (56%) cases directly treated COVID-19 patients in the isolation wards of COVID-19. However, there was no association between exposure to SARS-CoV-2 and immediate treatment of COVID-19 patients in isolation wards. In addition, some health workers did not wear the PPE level 4, including coveralls, gloves, face shields, boots, or N95 medical masks, outside the isolation ward. Most wore the surgical mask, head cap, glove, or fabric medical gown outside the isolation ward. Almost all medical staff wore masks at work in the hospital, even when they did not work in the isolation ward treating COVID-19 patients. The types of masks worn by the participants are presented in Table 2. Most of them wore N95 masks (44.6%) and KN95 masks (27.2%). In the univariate analysis, the number of PPE was not associated with the risk of infection. However, KN95 and N95 were associated with risk reduction of contracting COVID-19 infection, as compared to a single surgical mask (adjusted RR 0.27, 95% CI: 0.07, 0.97, *P* = 0.045 and adjusted RR 0.25, 95% CI: 0.07, 0.87, *P* = 0.029, respectively).

 Another factor that may influence the risk of contracting COVID-19 infection was compliance with health protocols inside or outside the hospital during the COVID-19 pandemic. The univariate analysis for the association between adherence to health protocols and the risk of COVID-19 infection is illustrated in Table 3. Most participants answered “always” (90.8%) to observing a physical distance of at least two meters during their activities. Compared to those who only sometimes observed physical distancing, there were no significantly increasing odds of contracting COVID-19 infection. In a similar vein, most HCWs also answered that they always washed their hands during activities (94.6% of participants), while there was no increased risk of being exposed to the SARS-CoV-2 virus, compared to those who only sometimes washed their hands. Outside their workplaces, only 63% of participants always wore masks during their activities. On the other hand, 27.7% and 9.2% of subjects, respectively, answered “sometimes” and “never” to the question of wearing a mask during activities when not handling the patients. The univariate analysis demonstrated an increased risk of contracting COVID-19 infection in subjects that sometimes and never wore masks during activities, compared to those who often wore masks (adjusted RR 2.36, 95% CI: 1.15, 4.85,* P* = 0.019 and adjusted RR 3.69, 95% CI: 1.27, 10.75, *P* = 0.017, respectively).

**Table 3:** Univariate analysis for the association of compliance to health protocols, exercise, and nutritional habits with the risk of COVID-19 infection after vaccination

**Variables**	**Frequency**	**Crude**		**Adjusted**	
		**RR (95% CI)**	***P*** ** value**	**RR (95% CI)**	***P *** **value**
Practice physical distancing					
Often	167	1.00			
Sometimes	17	1.09 (0.36, 3.24)	0.883	1.35 (0.44, 4.17)	0.600
Never	0	N/A		N/A	
Washing hand habit					
Often	174	1.00			
Sometimes	10	2.35 (0.65, 8.45)	0.192	2.71 (0.69, 10.52)	0.150
Never	0	N/A		N/A	
Wearing mask during activities					
Often	116	1.00			
Sometimes	51	2.31 (1.14, 4.67)	0.020	2.36 (1.15, 4.85)	0.019
Never	17	3.71 (1.30, 10.55)	0.014	3.69 (1.27, 10.75)	0.017
Regular exercise					
Yes	78	1.00			
No	106	1.09 (0.58, 2.05)	0.787	1.10 (0.56, 2.14)	0.792
Duration of exercise					
0–10 min/week	103	1.00			
11–149 min/week	64	0.88 (0.29, 2.72)	0.829	0.85 (0.27, 2.65)	0.773
≥ 150 min/week	17	0.89 (0.46, 1.76)	0.750	0.87 (0.43, 1.75)	0.689
Frequency of meal					
3 Times or more/day	87	1.00			
2 Times/day	89	1.14 (0.22, 6.06)	0.875	0.97 (0.17, 5.42)	0.973
1 Time/day	8	1.60 (0.31, 8.42)	0.577	1.36 (0.25, 7.56)	0.723
Fruit and vegetable consumption					
1 Portion/day (ref)	90	1.00			
1 Portion on 2-7 days	90	2.61 (1.35, 5.04)	0.004	2.73 (1.34, 5.57)	0.006
< 1 Portion on 1 week	4	1.25 (0.12, 12.66)	0.853	1.39 (0.13, 15.09)	0.786

N/A: not available.

 Our subsequent univariate analysis assessed the association of exercise and nutritional habits with the risk of COVID-19 infection after vaccination, as displayed in Table 3. Some participants did not do regular exercise in their routine (42.4% had regular exercise). Regarding duration, most of them only had 0-10 minutes of exercise in a week (56%). Despite that, regression analysis showed that the risk of COVID-19 infection was not linked to regular exercise and its duration. According to the frequency of meal history, 47.2% of the subjects had three or more meals per day, 48.4% of participants had two meals per day, and the other 4.3% had only one meal per day. No association was found between the frequency of meals and the risk of COVID-19 infection. In another survey, we asked about the frequency of fruit and vegetable consumption in their routine. Based on the univariate analysis, there was an increased risk of COVID-19 infection in subjects who consumed fruits or vegetables every 2-7 days, compared to those who had fruits or vegetables routinely every day (adjusted RR 2.73, 95% CI: 1.34, 5.57, *P* = 0.006).

 Multivariate logistic regression analysis is presented in Table 4. The crude multivariate analysis revealed that having contact outside the house or hospital, sometimes or never wearing masks during activities, and less vegetable consumption (1 portion on 2-7 days) increased the risk of COVID-19 infection after vaccination. On the contrary, wearing KN95 and N95 masks decreased the risk of acquiring SARS-CoV-2 infection. After adjustment for age, gender, and the presence of comorbidities, having contact outside the house or hospital (adjusted RR 6.82, 95% CI: 1.97, 47.98, *P* = 0.044), wearing KN95 (adjusted RR 0.06, 95% CI: 0.01, 0.51, *P* = 0.011) and N95 masks (adjusted RR 0.05, 95% CI: 0.01, 0.45, *P* = 0.007), never wearing mask during activities (adjusted RR 7.12, 95% CI: 1.88, 26.96, *P* = 0.004), and less fruit and vegetable consumption (adjusted RR 2.72, 95% CI: 1.12, 6.58, *P* = 0.027) were associated with the risk of COVID-19 infection after vaccination.

**Table 4:** Multivariate analysis for the variables associated with the risk of COVID-19 infection

**Variables**	**Crude**		**Adjusted**	
	**RR (95% CI)**	***P*** ** value**	**RR (95% CI)**	***P*** ** value**
Resident doctor	2.63 (0.62, 11.15)	0.189	5.01 (0.76, 32.94)	0.094
Contact with COVID-19 patients	1.00 (1.00, 1,00)	0.999	1.00 (1.00, 1.00)	1.000
Place of contact				
- House	1.31 (0.33, 3.89)	0.846	1.06 (0.29, 3.78)	0.934
- Outside house / hospital	7.73 (1.12, 53.9)	0.038	6.82 (1.97, 47.98)	0.044
Type of mask				
- KN95 mask	0.06 (0.01, 0.51)	0.010	0.06 (0.01, 0.51)	0.011
- N95 mask	0.06 (0.01, 0.49)	0.009	0.05 (0.01, 0.45)	0.007
Wearing a mask during activities				
- Sometimes	2.50 (1.01, 6.19)	0.048	2.39 (0.96, 5.97)	0.060
- Never	6.62 (1.81, 24.24)	0.004	7.12 (1.88, 26.96)	0.004
Fruit and vegetable consumption (1 portion on 2-7 days)	2.35 (1.01, 5.52)	0.049	2.72 (1.12, 6.58)	0.027

## Discussion

 Vaccination is one of the numerous strategies developed to prevent the transmission of SARS-CoV-2 infections. Nonetheless, previous studies revealed that the risk of COVID-19 disease might still exist despite receiving the immunization of CoronaVac fully. A national survey in Chile demonstrated that the effectiveness of CoronaVac vaccination in preventing the SARS-CoV-2 infection was 65.9% (95% CI 65.2% to 66.6%).^7^ Another study in Turkey reported that the efficacy of CoronaVac in preventing COVID-19 was 83.5% (95% CI 65.4-92.1%).^11^ The present study’s findings also revealed that the CoronaVac could protect 69% of participants against SARS-CoV-2 infection in the six months of follow-up. These findings strengthen the evidence that the odds of having a vaccine breakthrough still existed in subjects who had already received the two doses of the vaccine.

 In this study, several factors contributed to the increased risk of COVID-19 infection after vaccination. Resident doctors had an increased risk of SARS-CoV-2 disease, compared to specialists in our hospital. Along the same lines, Breazzano et al demonstrated that at least one resident with confirmed COVID-19 was reported in 45.1% of the residency program. In addition, 101 resident physicians were COVID-19 positive in New York City.^12^ The trainees who cared for more COVID-19 patients were more likely to test positive for COVID-19 and develop burnout syndrome.^13^ However, another study by Collins et al. revealed that anxiety and burnout in residents were not associated with a higher risk of COVID-19 exposure.^14^ Although there was still an unclear relationship between the risk of infection and residents, resident doctors were the frontline workers who treated COVID-19 patients in many University Hospitals in Indonesia. Working in a high-risk environment could increase the risk of COVID-19 infection.^15,16^

 The findings of this study pointed out that close contact with COVID-19 patients, especially outside of hospital or home, increased the risk of contracting COVID-19 infection. Eyre et al. also documented that hospital staff who had household contact with suspected or confirmed COVID-19 patients were more likely to acquire SARS-CoV-2 infection.^17^ The higher probability of SARS-CoV-2 infection in the community can be ascribed to inefficient use of PPE by HCWs. Most COVID-19 cases in Indonesia were underdiagnosed due to the lack of testing quantities.^18,19^ Therefore, this might have caused a higher odd of contracting SARS-CoV-2 infection in the community. HCWs who treated patients with COVID-19 did not show any differences in the risk of being infected with SARS-CoV-2, compared to those who did not. All HCWs who attended the COVID-19 wards wore PPE according to our hospital’s WHO and CDC guidelines. Several studies also indicated that adequate PPE could significantly protect HCWs against COVID-19 infection.^20,21^

 Outside the COVID-19 wards, not all HCWs adhered to the health protocols. Moreover, poor adherence to wearing masks when not treating COVID-19 patients increased the risk of acquiring SARS-CoV-2 infection. Regarding the type of mask, N95 and KN95 masks provided significant protection against SARS-CoV-2 infection, compared to a single surgical mask. Currently, no direct evidence exists on the effectiveness or comparative effectiveness of various respirators or masks in the prevention of SARS-CoV-2 infection in community settings. A meta-analysis study demonstrated similar protection provided by medical masks and N95 masks against this viral respiratory illness. Nevertheless, they did not compare the efficacy of these masks in preventing SARS-CoV-2 infection.^22^ The CDC had recommended using N95 and KN95 in non-aerosol-generating procedures to prevent SARS-CoV-2 infection.^23^ Diakonoff et al also demonstrated that HCWs, especially dentists, should wear (K)N95 masks during non-aerosol generating procedures.^24^ Despite that, several guidelines discouraged the use of N95 masks in community settings due to possible harms caused by improper use and the global shortage of N95 respirators.^23,25-26^

 Exercise during a pandemic is of utmost importance in preventing the health risks associated with physical inactivity. Immune activation against the infection induced by the exercise had been reviewed previously.^27^ However, a direct connection between exercise-induced immune changes and infection risk, especially against SARS-CoV-2, has not yet been established. The previous meta-analysis indicated that exercise did not reduce the number of acute respiratory infection episodes. Nonetheless, exercise reduced acute respiratory infection symptoms and the number of symptom days.^28^ Consistent with the current study, the stated research did not demonstrate any difference in the risk of SARS-CoV-2 infection between subjects who had regular exercise.

 Furthermore, it was found that healthy nutritional habits, including diets rich in fruit and vegetable, could reduce the risk of COVID-19 infection. Although the role of fruit and vegetable in the risk of SARS-CoV-2 infection is still unclear, Ponzo et al reported that lower adherence to a fruit and vegetable diet was associated with a higher risk of severe COVID-19.^29^ In another study, healthy diets containing a more frequent intake of fruits, vegetables, and fish were associated with a lower likelihood of SARS-CoV-2 infection.^30^

 In conclusion, we reported that the chance of SARS-CoV-2 infection still exists despite being fully vaccinated. This study provides new insight into the associated risk factors of vaccine breakthroughs in the Indonesian population. Wearing masks during activities, using a standardized mask (such as KN95 or N95 masks), and eating a healthy diet are still required to prevent the risk of SARS-CoV-2 infection. Nevertheless, this study suffers from some limitations. According to the design of this study, there might be a chance of recall bias when asking participants using the questionnaire. In addition, this study only demonstrated that several factors might be associated with the incidence of vaccine breakthroughs among health care workers; however, no causal conclusion could be reached due to the design of the study. Although we already randomized the participants enrolled in this study, there was still a chance of selection bias. All HCWs were recruited from a single hospital with similar demographic characteristics that would minimize the risk of selection bias in this study. However, since this study was conducted in a single-center hospital with a limited size of participants, great caution should be exercised in generalizing the results to other populations. Therefore, it is suggested that future studies be conducted using a multi-center approach and more participants in order to overcome this limitation.

## Acknowledgments

 The authors thank the Saiful Anwar General Hospital Malang, Indonesia, for the funding of this study. We thank the director of Saiful Anwar General Hospital Malang, Indonesia, Dr. Kohar Hari Santoso, SpAn, KAP, KIC for supporting this study. The authors also would like to thank dr. Mochamad Bachtiar Budianto, SpB(K)Onk as the assistant director of Education and Professional Development in Saiful Anwar General Hospital, Malang, Indonesia, and dr. Syaifullah Asmiragani, SpOT(K), as the assistant director of Medical and Nursing Services of Saiful Anwar General Hospital, Malang, Indonesia. The authors also thank Mohamad Fahmi Rizki Syaban, MD, for the assistance in writing the manuscript.

## Conflict of interest

 The authors declare that they have no conflict of interest.

## Funding

 None.

**Highlights**

- Healthcare workers (HCWs) are prone to the SARS-CoV-2 infection in the hospital despite being fully vaccinated.
- This study aimed to address the factors associated with COVID-19 vaccine breakthrough among HCWs.
- Wearing masks during activities, using a standardized mask (such as KN95 or N95 masks), and eating a healthy diet were still required to prevent the risk of SARS-CoV-2 infection among HCWs.
